# Single cell RNA sequencing improves the next generation of approaches to AML treatment: challenges and perspectives

**DOI:** 10.1186/s10020-025-01085-w

**Published:** 2025-01-30

**Authors:** Zahra Khosroabadi, Samaneh Azaryar, Hassan Dianat-Moghadam, Zohreh Amoozgar, Mohammadreza Sharifi

**Affiliations:** 1 Department of Genetics and Molecular Biology, School of Medicine, Isfahan University of Medical Sciences, Isfahan, 8174673461 Iran; 2 Pediatric Inherited Diseases Research Center, Isfahan University of Medical Sciences, Isfahan, Iran; 3 Edwin L. Steele Laboratories for Tumor Biology, Department of Radiation Oncology, Massachusetts General Hospital and Harvard Medical School, Boston, MA USA

**Keywords:** AI model, AML, Drug resistance, Single cell RNA sequencing, Tumor heterogeneity

## Abstract

Acute myeloid leukemia (AML) is caused by altered maturation and differentiation of myeloid blasts, as well as transcriptional/epigenetic alterations, all leading to excessive proliferation of malignant blood cells in the bone marrow. Tumor heterogeneity due to the acquisition of new somatic alterations leads to a high rate of resistance to current therapies or reduces the efficacy of hematopoietic stem cell transplantation (HSCT), thus increasing the risk of relapse and mortality. Single-cell RNA sequencing (scRNA-seq) will enable the classification of AML and guide treatment approaches by profiling patients with different facets of the same disease, stratifying risk, and identifying new potential therapeutic targets at the time of diagnosis or after treatment. ScRNA-seq allows the identification of quiescent stem-like cells, and leukemia stem cells responsible for resistance to therapeutic approaches and relapse after treatment. This method also introduces the factors and mechanisms that enhance the efficacy of the HSCT process. Generated data of the transcriptional profile of the AML could even allow the development of cancer vaccines and CAR T-cell therapies while saving valuable time and alleviating dangerous side effects of chemotherapy and HSCT in vivo. However, scRNA-seq applications face various challenges such as a large amount of data for high-dimensional analysis, technical noise, batch effects, and finding small biological patterns, which could be improved in combination with artificial intelligence models.

**Supplementary Information:**

The online version contains supplementary material available at 10.1186/s10020-025-01085-w.

## Highlights

- Tumor heterogeneity and drug resistance reduce therapeutic efficacy in AML treatment.
- ScRNA-seq improves the accuracy and quality of HSCT, resulting in a durable antitumor response.
- ScRNA-seq introduces neoantigens, biomarkers and cell subtypes that may contribute to reversing resistance to standard therapies in AML.
- Combination approaches using scRNA-seq and AI may provide effective AML management in the future.

**Supplementary Information:**

The online version contains supplementary material available at 10.1186/s10020-025-01085-w.

## Introduction

Acute myeloid leukemia (AML) is a hematologic malignancy of the myeloid lineage where disruption of myeloid lineage progenitor cells differentiation lead to uncontrolled leukemic blast proliferation in the bone marrow (BM), hindering the production of healthy blood cells (Thomas et al. 1977). In AML the annormally differentiated blood components originates in the BM but quickly infiltrates the bloodstream and can occasionally spread to testes, spleen, liver, lymph nodes, and central nervous system (Prada-Arismendy et al. 2017). Risk factors like aging, exposure to ionizing radiation or chemotherapy, obesity, smoking, and familial predisposition also contribute to the disease’s development (Poynter et al. 2016). AML is more common in the elderly, with a typical age of diagnosis around 67 years, who are more likely to have a lower performance status, more comorbidities, and be unfit. In the elderly, less than 25% of AML patients aged 60–65 survive for 5 years and less than 10% of those over 70 years old survive, compared to roughly 50% for patients under 50 years old (Wetzler et al. 2014).

Despite development of immunophenotyping- and cytogenetic -based prognostic biomarkers, AML remains a disease with a highly variable prognosis and a high mortality rate. Less than 50% of patients survive for 5 years overall, and only 20% of elderly patients survive to 2 years after diagnosis (Prada-Arismendy et al. 2017; Gupta and Sachs 2017). A number of therapeutic approaches have shown promise in improving clinical prognosis and extending survival, including the “3 + 7” regimen, allogeneic hematopoietic stem cell transplantation (HSCT), and molecularly targeted drugs that treat specific gene mutations and epigenetic abnormalities (Rowe 2022). Epigenetic abnormalities are heritable mutation that alters gene expression but has no effect on DNA sequence. Recent advances have introduced targeted medications, such as gilteritinib, midostaurin and astyrosine kinase inhibitors for targeting *FLT3* mutations, and ivosidenib and enasidenib for targeting mutant *IDH1* and *IDH2* (DiNardo and Wei 2020). Intensive chemotherapy achieves complete remissions in up to 70% of patients (Rollig et al. 2020). However, the complex pathophysiology of AML, high recurrence rates, patient age-dependent overall survival (OS) at time of diagnosis, and the variable genetic characteristics of blast cells limit the efficacy of treatment and the chance of survival (Bhansali et al. 2023; Dohner et al. 2022; Sasaki et al. 2021). The current standard of care options for patients deemed who are not candidates for intensive therapy offer poor 5-year overall survival rates (Rollig et al. 2020; Sasaki et al. 2021; Rausch et al. 2023; DiNardo et al. 2020a), highlighting the urgent need for more effective and less toxic treatments. The cell-based therapies utilizing allogeneic stem cells and donor lymphocyte infusions, followed by immunotherapy with monoclonal antibodies and chimeric antigen receptor (CAR) T cells, have revolutionized cancer treatment (Winer and Stone 2019). The FDA has approved an experimental new drug application (CB-012) for allogeneic anti-CLL-1 CAR T-cell therapy as a treatment for patients with relapsed/refractory AML (Biosciences 2023). However, low neoantigen expression and mutational burden, along with difficulties in identifying target antigens exclusively expressed on AML blasts, limit the effectiveness of treating AML (Daver et al. 2021; Haubner et al. 2019).

Tumor heterogeneity refers to when a primary tumor (intra-tumor) or tumors within and between the same histopathological subtype (inter-tumor), contain subpopulations of cells with different genotypes and phenotypes that may harbor divergent biological behaviors (Fisher et al. 2013). Understanding heterogeneity in cancer cells at transcriptomic level may improve future therapies. Single-cell RNA sequencing (scRNA-seq) enabels the examination of cell-specific gene expression patterns, their microenvironment, and their dynamic interactions, providing insights into tumor biology and heterogeneity, stress responses, treatment resistance, and biomarker identification (Zou et al. 2023; Shuang Geng1 et al.. 2019). Flow cytometry is commonly used to study cell heterogeneity in leukemia, while scRNA-seq is becoming increasingly important in the clinical diagnosis of hematologic malignancies, particularly focusing on the stem cell compartment (Zeijlemaker et al. 2019). Prognosis prediction models based on immune subtypes and gene expression patterns offer valuable insights for AML patient outcomes (Lu et al. 2022). Recent scRNA-seq studies on AML have revealed a variety of cells types, including primitive cells and differentiated cells, which may be linked to certain genomic features (Ediriwickrema et al. 2023). Researchers into primitive stem cell signatures has shown a correation with poor survival and differentiated AML cells may influence the immune system (Galen et al. 2019). It is now evident that different types of AML respond differently to new treatments, such as hypomethylating drugs like azacitidine and the oral BCL2 inhibitor venetoclax (Ediriwickrema et al. 2023). Recent studies have also identified potential therapeutic targets, such as CCAN1 and RAB37, for AML treatment. Functional precision medicine tumor boards have utilized scRNA-seq data to inform clinical decisions (Wu et al. 2020; Malani et al. 2022). The VIALE-A trial found that venetoclax plus azacitidine improved survival in older AML patients who were not good candidates. This is one of the few instances where observations made at the bench have translated into revolutionary practice, providing a successful treatment for a high-risk patient population with few therapeutic options (Ediriwickrema et al. 2023). However, resistance remains an issue, with some patients relapsing. Researchers use scRNA-seq and surface marker profiling simultaneously to investigate how resistance worked in patients who relapsed after treatment with azacitidine and venetoclax (DiNardo et al. 2020b). The discovery of these properties by scRNA-seq may provide a more efficient treatment than standard therapeutic approaches, as discussed in this review. Additionally, we present the challenges within scRNA-seq and then introduce its potential in combination with artificial intelligence to introduce more precise AML therapy in the era of personalized therapy.

## Treatment options and challenges for AML treatment

### Hematopoietic stem cell transplantation (HSCT)

HSCT involves autologous transplantation using the patient’s own stem cells or allogeneic transplantation from a donor, and is often used to treat hematologic disorders like AML (Hatzimichael and Tuthill 2010). A lack of knowledge about histocompatibility antigens led to mismatches between donors and patients (E D THOMAS HLLJ et al.. 1957), and increasing comorbidities and higher disease risks for relapse have been correlated with worse survival rates (Sorror et al. 2011). Allogeneic HSCT (allo-HSCT) improves survival rates for patients with AML and ables to regenerate a new hematopoietic system by replacing damaged BM tissue with healthy stem cells (Chen et al. 2023; Pohlmann et al. 2023). Allo-HSCT is the most widely used for myeloid malignancies for over 50 years. Allo-HSCT in AML patients has some limitations, including splenomegaly, graft-versus-host disease (GvHD), graft failure, infection, and immunosuppression. Strategies encompass sophisticated prophylactic medications, targeted T-cell depletion, and innovative immunomodulatory agents, all aimed at striking a delicate balance between averting GvHD and safeguarding the beneficial graft-versus-leukemia (GvL) effects (Malard et al. 2023; Choi et al. 2010; Cooke et al. 2017). Splenomegaly is associated with a poor prognosis and primary graft failure (Cheng et al. 2023; Ho and Becker 2013). Despite the belief that a larger spleen indicates a poor clinical prognosis, it is debatable whether spleen volume is independently linked to HSCT outcomes in AML patients. Older AML patients who underwent allo-HSCT had 3-year PFS and OS of 35% and 38%, respectively (Rashidi et al. 2016). Nevertheless, GvHD and infections linked to immunosuppression are the two major treatment-related problems that might arise following HSCT procedures (Pohlmann et al. 2023; Kurosawa et al. 2013). High-dose pretransplant chemotherapy limited by toxicity to the host hematopoietic system (D W BARNES MJC et al.. 1956). The identification of a suitable donor, especially for allogeneic transplants, poses a significat chellenge due to the strict criteria for genetic compatibility (Fleischhauer et al. 2023). Disease relapse, especially in the context of residual disease pre-transplant are exist, which could be allveited by CAR T-cell therapy or post-transplant maintenance therapies (Bader et al. 2023; Goldsmith et al. 2022; Goldsmith and Ghobadi 2022). Patients may encounter organ damage and the development of secondary cancers. Older patinets with AML may exhibit differences, leading to higher risk of chemotherapy resistance and a worse prognosis (Prassek et al. 2018). Many patients who are typically considered unsutiable for standard induction chemotherapy may not eligible for allo-HSCT (Megías-Vericat et al. 2018). Moreover, there is a lack of consensus on the best consolidation therapy for patients who achieve remission after induction treatment (Thol et al. 2015).

### Chemotherapy

Chemotherapy drugs like cytarabine/daunorubicin, gemtuzumab ozogamicin, decitabine, azacitidine, and clofarabine are commenly used to treat AML (Rosen et al. 2012). Anthracycline-based combination chemotherapy is the first choice for AML (Gupta and Sachs 2017). The “7 + 3” regimen, which involves cytarabine 100 mg/m2 for 7 days and idarubicin 12 mg/m2 for 3 days, is recommended treatment for newly diagnosed AML, achieving a 70% complete remission (CR) rate (Cheng et al. 2023). Patients who do not acheive CR after this regimen have a poor prognosis, with factors such as leukocytosis, older age, and high-risk cytogenetics contributing to induction failure (Ho and Becker 2013). Approximately 10–40% of newly diagnosed AML patients do not achieve CR with initial treatment and are classified as primary refractory (Thol et al. 2015). Additionally, over half of patients who initially achieve CR will eventually relapse (Megías-Vericat et al. 2018). As a result, chemoresistance and relapse are the main causes of death in AML (Huang et al. 2023). Induction failure in AML can be caused by a variety of factors including chemoresistance linked to DNA damage repair, cell quiescence, leukemic stem cell-related leukemogenesis, the TME, and therapy-induced clonal evolution (Cheng et al. 2023; Ng et al. 2016) (Fig. 3).

### Immunotherapy

#### Monoclonal antibodies

Monoclonal antibodies (mAbs) are artificially created molecules that specifically target proteins in cancer cells (Zahavi and Weiner 2020). CD38, a 45 kDa amino acid chain, is expressed in immune various cells, and is involved in lymphocyte signaling cascade that mediates activation, proliferation, and cytokine secretion (Williams et al. 2019). Leukemic stem cells (LSCs) can exist in the CD34 + CD38 + compartment of primary AML samples (Taussig et al. 2008). This target may be of relevance for AML, given that 75% of AML samples express CD38 and anti-CD38 mAbs with established safety profiles are approved for multiple myeloma (Williams et al. 2019). Immune checkpoint inhibitors (ICIs) are being studied or approved for AML treatment (Supplementary Table). Regards to the important roles of epigenetic in tumorigenesis and tumor progression, histone deacetylase (HDAC) inhibitors have been developed and show potential as a therapeutic approach, but they also upregulate PD-1 on T cells. Therefore, HDAC inhibitors should be used in combination with ICIs to target specific subtypes of AML for improved treatment outcomes (Radpour et al. 2021). Bispecific antibodies (BsAbs) such as have been developed to target two separate antigens simultaneously, such as the CLN-049 antibody binds to both FLT3 and CD3 (Mehta et al. 2022). Howver, antibody-based therapy have limited tumor penetration and retention rates (Wang et al. 2022), and most of them do not trigger apoptosis due to weak immunological responses in patients, particularly T-cell re-activation. The efficacy of mAbs is influenced by the amino acid sequence of the CH 2–3 domains and the Fc conserved glycan profile, which affect antibody dependent cellular cytotoxicity (ADCC) and complement-dependent cytotoxicity (CDC) activities (Jefferis 2009). Additionally, modifications in the TME, variability in target antigens, the phenotypic diversity among leukemic cells, and significat cross-talk between signaling pathways in cancer cells and surrounding cells contributes to reduction of therapeutic efficacy of mAbs in vivo relapses in their treatment (Brinkmann 2017; Torka et al. 2019). Therefore, understanding and overcoming the intricate dynamics of AML resistance mechanisms and the immunosuppressive TME is paramount for advancing the impact of mAb-based therapies (Renard et al. 2021).

#### CAR T-cell -based therapy

Chemeric antigen reseptor (CAR) technology, which involves modifying T lymphocytes to express the recombinant receptors that redirect their specificity and function within a single molecule to target cancer proteins specifically (Hafemeister and Satija 2019). While potent and approved in clinical trials in treating AML, the practical application of this personalized treatment presents logistical challenges and may not be universally applicable to all patients (Altman and Krzywinski 2018). CD93 is pioneering target in MLL-rearranged (MLLr) AML, and to address concerns about potential endothelial cell toxicity, the NOT-gated CARs were introduced to inhibit CAR T-cell activation in the presence of antigens in cross-reactive tissues (Sun et al. 2019). Additionally, the presence of T cells intensifies the risk of such damage (Kim et al. 2019). Other potentils issues associated with CAR T-cell therapies are including cytokine release syndrome (CRS), immune effector cell-associated neurotoxicity syndrome (ICANS), and on-target/off-target toxicities (Fig. 5). Overall, lack of specific antigens for AML lead to toxicities that can cause lasting damage to the BM and potentilly affect other healthy organs and tissues.

#### Cancer vaccine

AML vaccines leverage the immune system’s ability to recognize and eliminate specific antigens commenly found in leukemia (Kaczmarek et al. 2023). A novel approach to AML vaccination is AMCNP therapy (antigen-capturing and membrane-associated nanoparticles) that has shown significant improvements in OS rates, even when leukemia cells are reintroduced. AMCNP therapy is a flexible and multi-antigenic approach, eliminating the need for time-consuming and expensive identification of patient-specific neoantigens (Johnson et al. 2022). The ability of CpG-encapsulated AMCNPs to enhance the activation of dendritic cells (DCs), the expression of co-stimulatory signals, and the presentation of leukemic antigens suggests their potential for future use in clinical settings. Compared to traditional whole cell lysate (WCL) vaccines, AMCNP offers personalized treatment with multiple antigens and efficient delivery to DCs (Johnson et al. 2022). A phase I/II clinical trial and in a personalized tumor vaccine, patient-derived tumor cells are combined with autologous DCs showed that immunizations of AML patients increased the number of leukemia-specific T cells and many patients remained in remission after an average of 5 years of follow-up. To address T-cell repertoire dysfunction and the limited efficacy of checkpoint inhibitors, the study combines the this vaccine with ICI, which offering a more targeted and effective treatment option for AML patients (Stroopinsky et al. 2021). Aphase I/II clinical trial using Wilms’ tumor 1 recombinant protein (WT1-A10) and adjuvant AS01B in elderly AML patients after chemotherapy and showed CD4 + T cell immune responses, measurable disease clearance, and long-term molecular remission (Kreutmair et al. 2022). mRNA vaccines wre developed to identify appropriate individuals for vaccination. Analytical GEPIA2 and Tumor Immune Single-cell Hub techniques were employed to examine genes that are expressed differently and to determine the connection between putative tumor antigens and immune cells in BM. Researchers also utilize consensus clustering analysis to identify distinct immune subtypes, which five potential tumor antigens including CDH23, LRP1, MEFV, MYOF, and SLC9A9 associated with the infiltration of DCs (Wang 2023). However, developing cancer vaccines requires individualized vaccines tailored to specific tumor antigens (Kaczmarek and 1 et al.. 2023). In situ vaccination limit discovering specific tumor antigens due to genetic heterogeneity and the TME (Buonaguro and Tagliamonte 2020; Hammerich et al. 2015). Peptide-based cancer vaccines just focuse on specific antigens and and some cells may lack targeted peptides (Saxena et al. 2013). The requirement for peptides to bind to specific HLA molecules limits their use in certain patients, excluding a significant portion of the population. Tumor genetic diversity complicates the efficacy of peptide vaccine, resulting in suboptimal immune responses (Stephens et al. 2021).

## Single cell RNA sequencing

**Fig. 1:**
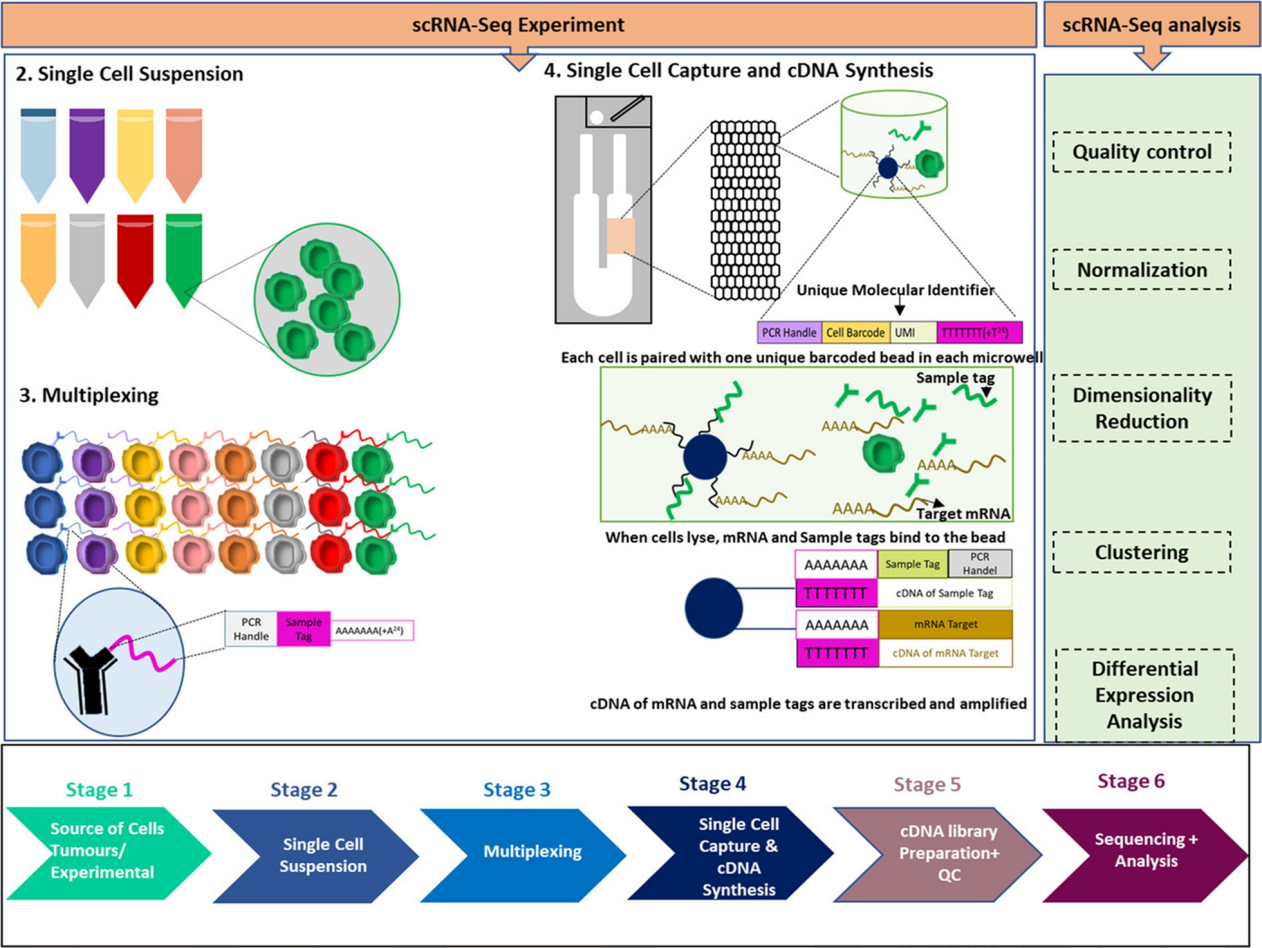
The scRNA-Seq pipeline involves multiple stages. At first, a live single-cell suspension is created from tissue samples or cell cultures and sorted. Each sample is then combined with a specific antibody and pooled. Next, single cells are lysed to release mRNA and labeled with a unique oligo sequence for cell identification. The synthesized cDNA and sample tags are then amplified to generate the library. After sequencing, the scRNA-Seq data is analyzed using a series of steps outlined in the figure. Reprinted from ref (Ke et al. 2022)

RNA-seq technology has enabled researchers to study intricate biological processes by mapping and quantifying transcripts in disease states (Fadl et al. 2020). However, traditional bulk RNA-seq only provides the average gene expression per tissue or cell culture, which hides the variability between individual cells and makes it challenging to analyze minor cell subpopulations. To address this limitation, scRNAseq has emerged as a revolutionary tool that allows for the exploration of gene expression profiles of thousands of cells at the single-cell level in just one experiment (Ramsköld et al. 2012; Macosko et al. 2015). A typical scRNA-seq pipeline consists of several steps: (**i**) sample acquisition; (**ii**) creation of a single cell suspension; (**iii**) multiplexing; (**iv**) single-cell capture and cDNA synthesis; (**v**) cDNA library preparation and quality control (QC); and (**vi**) sequencing and data analysis (Molin and Camillo 2019) (Fig. 1).

**Fig. 2:**
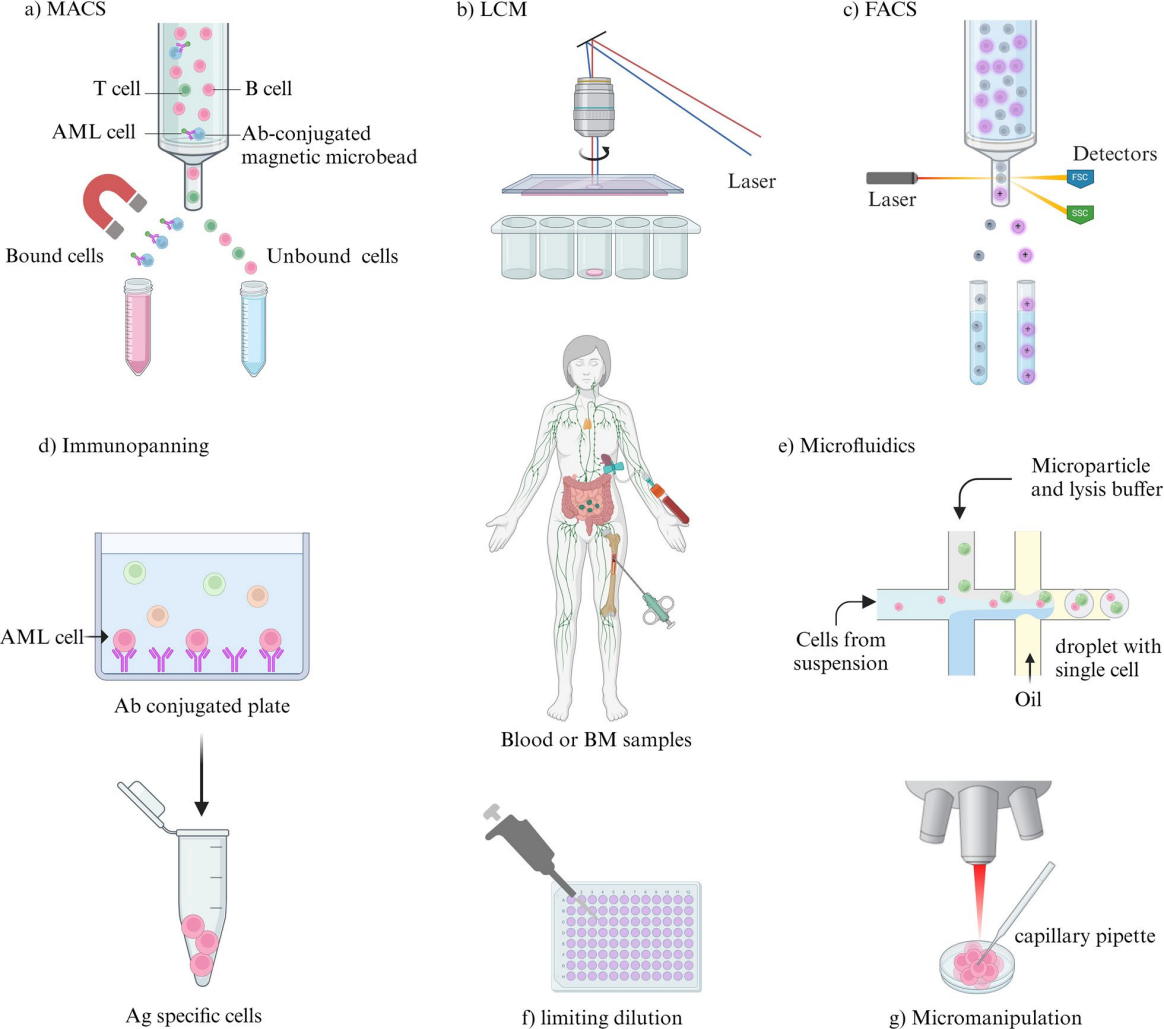
Overview of single-cell isolation technologies. **a **MACS: Magnetic beads conjuncted with a specific antibodies are used to label cells of interest. The labeled cells are then extracted from the cell solution using an external magnetic field. **b **LCM: This method separates cells from solid samples using laser equipment and a computer system. **c **FACS: Fluorescent marker proteins are used to tag cells, allowing for the isolation of highly pure single cells. **d **Immunopanning: This process involves incubating cells on an antibody-coated plate to capture the particular antigens on the cells. The bound cells are reatained while unbound cells are washed away. **e **Microfluidics: Microfluidic technology is used for single-cell separation, requiring nanoliter-sized volumes. An example of this is Drop-Seq, which utilizes in-house microdroplets. **f **Limiting dilution: This approach utilizes the statistical distribution of diluted cells to identify individual cells. **g **Micromanipulation: Single cells are collected using capillary pipettes guided by a microscope in micromanipulation

Capturing single cells with high efficiency is one of the major challenges of single-cell sequencing. Currently, there are several methods utilized to isolate single cells (Fig. 2), that among them, the microfluidics has become a popular method due to its low sample consumption, precise fluid control, and low operating costs (Qi et al. 2019). Droplet-based microfluidic (microdroplets) is currently the most popular high-throughput platform, in which single cells are masked by nanoliter droplets that contain a lysis buffer and barcoded beads using microfluidic and reverse emulsion devices (Choe et al. 2023). The Chromium System by 10X Genomics is microdroplets that is frequently utilized in utilizing scRNAseq on AML samples through “Chromium Next GEM Single Cell 3′ Reagent Kits v3.1.” (Madaci et al. 2023). Barcoded primer gel beads, oil-surfactant solution, and single cells with GemCode Single-Cell 3′ reagents are combined in 10X Genomics to form gel bead in emulsions, known as GEMs. Reverse transcription occurs within the GEMs, making their formation crucial for the success of scRNA-seq. Hydrogel beads are used in the 10X Genomics and inDrop systems, while brittle resin beads are used in the Drop-seq system. Because hydrogel beads are softer and more flexible than the small, hard brittle resin beads, the encapsulation of one hydrogel bead and one cell in the same droplet follows a super-Poisson distribution, whereas using brittle resin beads results in a double poisson distribution. After encapsulation, the primers in the 10X Genomics and inDrop systems are released into the solution phase, whereas in Drop-seq they are bound to the surface of the beads. As a result, the cell capture efficiency in the 10X Genomics and inDrop systems is significantly higher (Zhang et al. 2019). The 10X Genomics technique significantly lowers labor, cost, and processing time by combining several steps a single procedure. 10X Genomics is optimized for higher output and produces higher molecular sensitivity and precision with less technical noise (Chen et al. 2019a).

The data analysis section consists of five steps: (**i**) Quality control (QC) by FastQC for finding and eliminating low-quality cells due to scRNA-seq experiments may provide some low-quality data from cells that are damaged, dead, or mixed with multiple cells (Ilicic et al. 2016), which are leading to hinder downstream analysis and may lead to misinterpretation of the data (Chen et al. 2019b; Kiselev et al. 2017). (**ii**) Normalizationfot for removing technical effects that influence underlying molecular counts while preserving real biological variation is the primary goal of single-cell normalization. A successful normalization approach should be no correlation between the total sequencing depth of a cell and the normalized expression level of a gene. Sequencing depth variations should also not affect downstream analytical tasks such as dimensional reduction and differential expression. Regardless of gene abundance or sequencing depth, the variance of a normalized gene (across cells) should mainly reflect biological heterogeneity (Hafemeister and Satija 2019). (**iii**) Dimensionality reduction for scRNA-seq analysis as they convert the original noisy expression matrix, which is high-dimensional, into a low-dimensional subspace with enhanced signals (Altman and Krzywinski 2018) and thus, can help with data visualization, noise reduction, and the efficient and successful downstream analysis of scRNA-seq (Sun et al. 2019). (**iv**) Clustering for characterization of cell types and/or subpopulations based on their transcriptome signatures to discern cell identity and describe their functions (Kim et al. 2019). Following this, biological information, often derived from marker genes identified in published studies, is commonly used to precisely determine the cell type in each cluster and/or enhance the accuracy of clustering outcomes (Kim et al. 2019). (**v**) Differential expression analysis for identifying significantly differentially expressed genes (DEGs) when comparing distinct subpopulations or groups of cells. These DEGs are essential for understanding the biological differences between the compared conditions (Chen et al. 2019b).

Researchers must make several decisions when conducting scRNA-Seq based on the research question and sample complexity. These include choosing the appropriate platform, determining the desired number of cells and sequencing depth, and selecting computational analysis approaches. The decision on which scRNA-Seq platform to adopt will also be influenced by the required depth and coverage of the transcriptomic data (Ke et al. 2022; Gao et al. 2020). Usually, relevant procedures fall into two primary categories: those that involve sequencing the full-length transcripts and those that focus on the 3′ or 5′ ends of transcripts (tag-based methods) (Yang XSaH. Single-cell RNA sequencing to track novel perspectives in HSC heterogeneity. 2022). Compared to 3′-end or 5′-end counting protocols, full-length scRNA-seq methods have significant advantages in isoform usage analysis, allelic expression detection, and identifying RNA editing markers due to their superior transcript coverage. However, these methods are more expensive that based tag-based technology (Huang et al. 2023).

## Prospects of ScRNA-seq to improve the treatment of AML

### How scRNA-seq could improve AML chemotherapy?

**Fig. 3:**
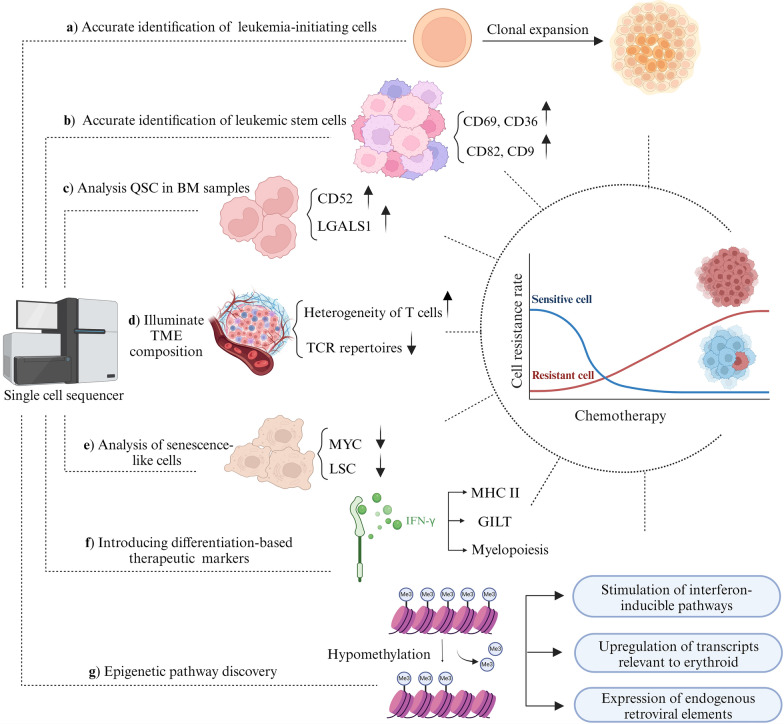
The potential role of scRNA-seq in the management of HSCT therapy in AML

ScRNA-seq provides the discoveries of (i) new cell subsets and markers, (ii) intertumor and intratumor heterogeneity, (iii) the TME, (iv) intercellular communication, and (v) lineage pathways, which all promote our understanding of AML pathogenesis, diagnosis, prognosis, treatment, relapse and chemoresistance (Fig. 3).

#### scRNA-seq and identification of leukemic stem cell-related chemoresistance

LSCs are an essential factor in chemoresistance in AML, originating from various cell types through distinct oncogenic drivers and pre-leukemic events (Vetrie et al. 2020). The concept of LSCs emerged from observations in NOD/SCID mouse engraftment trials, where leukemia could only be reconstituted by a subset of AML cells with an immature immunophenotype (CD34 +, CD38-) (Duy et al. 2021). Despite their genetic abnormalities, LSCs share similarities with normal HSCs asthey are quiescent, multipotent, and capable of self-renewal (Gupta and Sachs 2017). While most leukemia patients have rapidly dividing leukemia cells that respond well to first-line AML therapy, the self-renewing leukemia cells that survive can lead to relapse (Sachs et al. 2020). Genome analysis has revealed pre-leukemic mutations in HSCs that promote clonal expansion in AML and survive standard induction chemotherapy, contributing to disease relapse (Shlush et al. 2014). Thus, understanding the biology of LSCs and developing therapeutic strategies that specifically target them are essential for acheiving long-term AML remission (Gupta and Sachs 2017).

In normal HSC under steady-state conditions, the functions of self-renewal and proliferation are distinct. Antiproliferative treatments may fail to target self-renewal if these roles are also distinct in LSCs, which could lead to relapse. Sachs et al. (Liu et al. 2024) used scRNA-seq to identify unique transcriptional profiles in Mll-AF9/NRASG12V murine AML LSCs. They discovered two subgroups of AML LSCs in mouse models that expressed high levels of CD69 and CD36, respectively. The subpopulation with high CD69 had a greater potential for proliferation but was unable to initiate leukemia development, while the subpopulation with high CD36 demonstrated stronger self-renewal but was unable to proliferate. The LSC-specific genes CD69 and CD36 correlated with these markers in human samples, consistent with the mouse observations. However, these data do not definitively show whether CD69 and CD36 proteins can distinguish human LSCs with varying capacities for self-renewal, even though their levels correlate with varied proliferative rates in human AML. Future studies can use the CD36^High^ or CD69^High^-sorted human LSCs in leukemia xenografts or serial colony-forming tests and then introduce CD36 or CD69 for antibody-based targeted therapies (Sachs et al. 2020).

The expression of CD36 has been linked to changes in the metabolism and chemoresistance of leukemia cells (Ye et al. 2016; Farge et al. 2017); this observation aligns with a proleukemic effect. The glycoprotein cell surface receptor CD36 is involved in various processes, including platelet regulation, fatty acid transport, and cell adhesion. Another gene specific to LSCs in this analysis, *MYB*, has been linked to AML self-renewal, and with potential inhibitors currently in development. The results of this research indicate that LSCs cannot simultaneously self-renew and proliferate, suggesting that preventing AML relapse may require addressing both of these characteristics (Ramaswamy et al. 2018; Walf-Vorderwülbecke et al. 2018).

In a study conducted by Cheng et al. (2023), scRNA-seq was used to assess the genomic profiles of 28,950 AML cells from 13 AML patients. They found that chemoresistant AML cells accumulated prematurely during early hematopoiesis. Compared to the CR group, the non-CR group’s hematopoietic stem cell-like cells showed higher levels of LSC markers (e.g., CD9, CD82, IL3RA, and IL1RAP). Due to an early stoppage of hematopoiesis, chemoresistant progenitor cells exhibited poor myeloid differentiation (Cheng et al. 2023). Another study found that miR-126-high LSCs were abundant in chemotherapy-refractory AML at diagnosis and relapse and that these cells promoted chemotherapy resistance by exhibiting enforced stemness and quiescence features (Naldini et al. 2023).

#### scRNA-seq and senescence-related chemoresistance

Regardless of LSC status, another discovery by Duy et al. (2021) suggests that a robust subpopulation with a senescence-like phenotype mediates AML relapse. Chemotherapy causes initial AML cells to display senescence-like behavior both in vitro and in vivo. Additionally, transcriptional processes associated with senescence/inflammatory responses and embryonic diapause are activated, leading to downregulation of genes for LSCs and MYC. ScRNA-seq analysis revealed enrichment for subpopulations with unique senescence-like cells and a decrease in LSCs both in vitro and in vivo. This temporary senescence effect resulted in enhanced colony-forming and engraftment capacities.

The ataxia telangiectasia and Rad3-related protein (ATR) played a crucial role in facilitating AML cells to enter this senescence-like phase, and the use of ATR inhibitors significantly impaired the AML cells’ survival. Consequently, ATR inhibition emerges as a possible therapeutic strategy, suggesting that the enrichment of stem-cell characteristics at relapse may be a result of chemotherapy rather than a cause of chemotherapy resistance (Duy et al. 2021).

#### scRNA-seq and dormancy-related chemoresistance

ScRNA-seq technology used to analyze the cellular states in BM samples from primary refractory AML patients or those who relapsed rapidly after therapy. By tracking the dynamic transcriptional changes resulting from chemotherapy in AML patients, it was demonstrated that proliferating stem/progenitor-like cells (PSPs) could be reprogrammed to express similar quiescent stem-like cells (QSCs) (Duy et al. 2021). It was hypothesized that this cellular reprogramming induced by chemotherapy might be dose-dependent. PSPs would acquire the QSC-like transcriptional program under a treatment dose that would not kill them, making them more resilient (Bassan et al. 2019).

QSCs express the targetable markers CD52 and LGALS1, which are also overexpressed in PSPs from relapsed/refractory AML (RR-AML) patients and chemo-residual QSCs (Ruvolo et al. 2020). Further confirmation of the presence of chemoresistance-associated CD99 + CD49d + CD52 + Galectin-1 + QSCs at diagnosis was obtained through flow cytometric analysis. These cells were found to be concentrated in the residual AML cells of refractory individuals. The poor prognosis and immune evasion associated with AML may be influenced by the interaction between QSCs and monocytes via CD52-SIGLEC10. Additionally, researchers found that LGALS1 was a viable target for chemoresistant AML and an LGALS1 inhibitor can eradicate QSCs in patient-derived primary AML cells, cell lines, and AML xenograft models (Li et al. 2023). The results of this study will contribute to a better understanding of the mechanism behind chemoresistance in AML and to the development of new therapeutic strategies for patients with resistant or relapsed AML.

The heterogeneity of leukemia-initiating cells’ (LICs) poses a significant barrier to AML treatment. Research indicates that AML patients often have multiple types of LICs with varying leveles of pathogenicity. However, the funactional differences between these coexisting LICs, particularly in term of their response to teratment, remain unclear. To address this knowledge gap and potentially enhance treatment outcomes, a study was conducted (Song et al. 2021). Using scRNA-seq, researchers to identifyed two cell subsets, c-Kit + B220 + Mac-1 − and c-Kit + B220 + Mac-1 + , that were enriched for LICs in a murine Setd2-/- AML model. These subsets exhibited distinct chemotherapeutic responses and differentiation potentials. In vivo administration of doxorubicin and cytarabine to the c-Kit + B220 + Mac-1 + cells resulted in increased activation of the RAS pathway and intrinsic resistance. Combining these drugs and RAS pathway inhibitors resultes in the death of both types of LICs and reduced the progress of the disease (Song et al. 2021). These findings improve our knowledge of the intra-heterogeneity of LICs and may lead to development of more effective therapies.

#### scRNA-seq and epigenetic-related chemoresistance

The molecular mechanisms involved in response to decitabine in AML and myelodysplastic syndrome (MDS) are still not fully understood. The aim of this study investigating these mechanism using scRNA-seq. Total BM aspirate cells from 10 patients were collected on days 0 and 10 of decitabine treatment (Upadhyay et al. 2022). After 10 days of treatment, it was found that decitabine caused global, reversible hypomethylation in all patients. This ypomethylation was linked to the upregulation of transcripts relevant to erythroids, the expression of endogenous retroviral elements, and the stimulation of interferon-inducible pathways. Erythroid-related pathways were inhibited by the treatament, but upon relapse, they were restored (Upadhyay et al. 2022).

#### scRNA-seq and differentiation-related chemoresistance

IFN-γ caused overexposure or increased responsiveness in chemoresistant HSC-like cells and in some AML patients without CR the IFN-γ-inducible lysosomal thiol (GILT) was overproduced (Florez et al. 2020). This means that AML chemoresistance may be influenced by IFN-γ-induced GILT. Furthermore, IFN-γ induced MHC II antigen expression, processing, and presentation in non-CR HSC-like cells, providing more evidence for IFN-γ’s role in AML chemoresistance (Cheng et al. 2023; Niu et al. 2021). In addition to HSC-like cells, the HSC niche, which consists of diverse cell populations, is essential for preserving and controlling the HSC pool in AML chemoresistance (Sánchez-Aguilera and Méndez-Ferrer 2017; Jeong et al. 2018). Terminal differentiation may be affected in the bone marrow by the replacement of normal HSCs with leukemic niches (Kim et al. 2015). The non-CR group of HSCs may interact with cytotoxic CD8 + T cells, an IFN-γ signaling source, to promote myeloid cell accumulation and enhance myelopoiesis. This indicates that chemoresistant AML cells undergo hematopoietic arrest earlier than chemosensitive AML cells (Schürch et al. 2014). To help patients with AML overcome the stocked differentiation process, differentiation therapy may be an alternative approach.

The success of all-trans retinoic acid (ATRA) in treating acute promyelocytic leukemia (APL) has shown the potential of differentiation therapy to improve survival in AML patients. However, this success has not been replicated in other subtypes of AML (Takahashi 2021). Therefore, there is a need to discover new therapeutic agents that can induce differentiation in AML blasts. Additionally, it is widely recognized that epigenetic changes play a crucial role in the progression and persistence of cancer, especially in AML. To induce differentiation in AML, Edurne San Jose et al. studied the development of novel small molecules that specifically target epigenetic modifying enzymes, including DNA methyltransferases (DNMT), histone methyltransferases, or HDAC. They screened over fifty tiny compounds that their group had produced for this study. The screening was predicated on alterations in the expression of CD11b, a well-characterized marker of myeloid differentiation, following the in vitro treatment of AML cell lines. From the results of this investigation, CM-444 and CM-1758 were chosen as the lead compounds because of their great capacity to induce leukemic cell differentiation in AML cell lines at low, non-cytotoxic dosages. After thorough biochemical analysis, it was shown that both substances are particular pan-HDAC inhibitors (HDACi). Independent of AML genetic subtypes or the presence of mutations, CM-444 and CM-1758 induced cell differentiation in vitro in all AML subtypes. This differentiation was noticeably stronger than that induced by reference compounds like Panobinostat, Vorinostat, Entinostat, Tubastatin, or Quisinostat, an HDACi that has been described previously. Additionally, in vivo differentiation was triggered in AML xenogeneic models by CM-444 and CM-1758. AML differentiation was linked to morphological alterations, downregulation of c-MYC, overexpression of transcription factors controlling myeloid differentiation, and induction of CD11b. These substances also encouraged the in vitro differentiation of AML blasts obtained from patients (San Jose et al. 2019). Apart from ATRA, CM-444 and CM-1758 which are used to differentiate acute promyelocytic leukemia cells, inhibitors of FLT3 and isocitrate dehydrogenase 1 also cause AML cells to undergo apoptosis, leading to cell differentiation (Martinez-Høyer et al. 2020; Madan and Koeffler 2021). A potential benefit of using differentiation agents to treat AML may be less toxicity and more clonal selection (Stubbins and Karsan 2021).

#### scRNA-seq and clonal expansion -related chemoresistance

It is crucial to understand the changes in leukemia composition following medication, particularly at the cellular level. Medication exerst selective pressure on a robust subgroup, leading to clonal expansion, selection, genetic mutation, and accumulation, all of which are intrinsically linked to resistance (Greaves and Maley 2012). Single-cell sequencing technology was used to investigate the causes of drug resistance resulting from therapy-induced clonal expansion and evolution of tumor cells. The persistence of LICs after AML treatment is considered to be the cause of relapse. Using scRNA-seq, Stentson et al*.* (2021) were the first to evaluate RNA-based changes in LICs from matched patient diagnosis and relapse samples in AML, where the hierarchical structure is well established based on DNA sequence. They also identified LICs that undergo RNA clonal evolution during AML progression. Pathway analysis revealed shared signaling networks involving metabolism, apoptosis, and chemokine signaling that developed during AML progression and became characteristic of relapse samples, despite the presence of significant transcriptional variation at the single-cell level. According to this research, curative treatment will need to target multiple pathways simultaneously. This is demonstrated through targeting BCL2 and CXCR4 signalings, resulting in the eradication of LICs and enhancing the treatment response in preclinical model (Stetson et al. 2021).

#### scRNA-seq and TME -related chemoresistance

TME plays a crucial role in leukemia development and could be a potential targets for treatment. By analyzing TME, particularly immune cells, single-cell sequencing investigations have recently contributed to the understanding of drug effects and resistance mechanisms (Korn and Méndez-Ferrer 2017; Skelding et al. 2022). While ICI has shown better results in solid malignancies, its impact on AML treatment has been modest at best (Daver et al. 2019). Two studies utilized single-cell TCR sequencing, immunological profiling, scRNA-seq, and CITE-seq on AML patients treated with a combination of hypomethylating drugs and PD-1 inhibitors to explore the causes of resistance to ICI (Goswami et al. 2022; Abbas et al. 2021). Pre/post azacytidine + nivolumab treatment, paired single-cell RNA analysis, and TCR repertoire profiling of BM cells in patients with relapsed/refractory AML demonstrated the high heterogeneity of disease-related T cell subsets and the changes in their abundance that occur with PD-1 blockade-based treatment. Patients responding to treatment or in a stable state of their illness, showed an increase in TCR repertoires originating mostly from CD8 + cells;, while n non-responding patients exhibited a decrease in TCR repertoires. Trajectory analysis revealed a continuum of CD8 + T cell phenotypes distinguished by the differential expression of granzyme B and a memory CD8 + T cell fraction that resides in the BM and is enriched in responders, expressing granzyme K. Additionally, chromosomal 7/7q deletion was identified as an intrinsic cancer genomic marker of resistance to PD-1 treatment in AML using single-cell-based CNV analysis (Goswami et al. 2022; Abbas et al. 2021).

Since deregulation of epigenetic modifications is a hallmark of AML and plays a role in the disease’s pathogenesis, preclinical and clinical studies on leukemia have primarily focused on targeting the epigenome through the pharmacologic inhibition of epigenetic modifiers (Gambacorta et al. 2019). Epigenetic agents can impact the anticancer immune response by controlling immune cell function in the TME (Aspeslagh et al. 2018). Salmon et al. discovered a novel immunoregulatory mechanism through the inhibition of HDAC using CITE-seq and scRNAseq. Activation of the IFN I pathway was crucial for leukemia cell differentiation and the therapeutic efficacy of panobinostat, an HDAC inhibitor, in models of AML. Administration of panobinostat leds to generation of IFN I by plasmacytoid dendritic cells (pDC) via enhancing H3K27 acetylation at IFN gene loci. Reduced pDC levels hindered the induction of type I IFN signaling in leukemia cells mediated by Panobinostat, minimizing treatment efficacy. Conversely, combined treatment with panobinostat and IFNα improved results in clinical models by augmenting pDC counts and enhancing H3K27ac near the IFNα loci, therefore boosting IFN I production (Salmon et al. 2022).

### How scRNA-seq could improve HSCT to treat AML?

The examination of scRNA-seq is crucial for understanding the dynamics of immune cell populations and hematopoietic stem and progenitor cells (HSPCs) before and during transplantation, even if it is not directly related to HSCT (Buonaguro and Tagliamonte 2020). scRNA-seq can define the composition of the hematopoietic graft, including various cell types and developmental stages, prior to transplantation. This information aids in evaluating the graft’s quality and predicting its likelihood of engraftment and long-term reconstitution (Hammerich et al. 2015).

Konturek-Ciesla et al. investigated the impact of aging on the hematopoietic system, focusing on HSCs. Utilizing native long-term lineage tracing in mice, the researchers observed altered kinetics in mature blood cell production from aged HSCs, with a preference for platelet differentiation. scRNA-seq plays a crucial role in identifying new cell populations during early hematopoiesis in aging, emphasizing a differentiation block in aged HSCs. The study recognized that aging is associated with both intrinsic changes as well as extrinsic factors such as niche remodeling and increased inflammation. Exploring rejuvenation strategies, in vitro HSC expansion protocols were tested, revealing viability but a failure of ex vivo expanded aged HSCs to support stable multilineage hematopoiesis. However, increasing the input number of aged HSCs improved reconstitution, indicating heterogeneity and the presence of rare clones capable of efficient lymphopoiesis. The study concludes by highlighting the opportunity to explore and understand lymphoid-competent aged HSC clones in vitro, aiming to reinstate healthier hematopoiesis in aging individuals (Konturek-Ciesla et al. 2023).

Novel options for evaluating stem cell heterogeneity are currently provided by scRNA-seq, enabling extensive gene expression profiling at the single-cell level (Saxena et al. 2013). Moreover, scRNA-seq has been useful in the optimization of HSCT in AML. scRNA-seq can identify the subpopulations of immune cells associated with treatment outcomes after transplantation. For instance, Mathioudaki et al. (2024) used scRNA-seq to study T cells in the BM of 6 patients with AML who had undergone allogeneic HSCT and found T-cell signatures that were different for patients who were in remission or relapse. Patients who were in remission had more cytotoxic CD8 + T cells, while those who were at risk of relapse had an immunosuppressive T-cell environment that included factors like TNF/NF-κB and other inflammatory signaling pathways. This showed that scRNA-seq could be used to predict relapse and guide personalized treatments in order to improve HSCT outcomes. Other studies have also described how scRNA-seq could enhance the GvL effect with reduced GvHD. Researchers have used scRNA-seq to profile immune responses in patients receiving combination therapies, such as azacitidine with immune checkpoint inhibitors (ClinicalTrials.gov identifier: NCT02397720). This has demonstrated capabilities in the stratification of patients toward specific post-transplant interventions to improve therapeutic efficacy. Moreover, the discovery of biomarkers such as GPR56 on CD8 + T cells associated with improved survival further illustrates the capability of scRNA-seq to monitor immune recovery and optimize donor cell selection (Root et al. 2024). This will open up avenues for better stratification and personalized treatment approaches in AML.

In another study, Pellin et al. (2019) utilized scRNA-seq to analyze the healthy donor BM CD34 + cells, revealing a hierarchically-structured transcriptional landscape of hematopoietic differentiation. Their study offered a comprehensive transcriptional profiling of lineage-negative hematopoietic progenitors in the BM, uncovering a crucial branch point leading to basophils. This expanded our understanding of early adult human hematopoiesis. Importantly, the research demonstrated strong similarities in topology and gene expression between human and mouse hematopoiesis. This study identified CD164, a sialomucin, as a reliable marker for the earliest stages of HSPC specification. The use of CD164 as a marker was shown to enhance the design of alternative transplantation cell products (Pellin et al. 2019). This data addresses issues with graft failure or low quality and helps predict the chance of engraftment and long-term reconstitution (Stephens et al. 2021).

With scRNA-seq, researchers can monitor transcriptional changes occurring during engraftment and reconstitution after HSCT. To address issues like GvHD and disease relapse, understanding molecular dynamics at the single-cell level is helpful, providing important insights into immune system dynamics and hematopoietic recovery (Fadl et al. 2020). Single-cell definitions are used to describe HSCs, but there are differences in the multipotent progenitor pool. Accurate assessment of cell function at both the population and individual cell levels is necessary for research on HSPCs. A better understanding of these cells’ behavior may enable researchers to create focused therapies to address issues like GvHD and recurrence (Buonaguro and Tagliamonte 2020; Molin and Camillo 2019).

scRNA-seq enables clinicians to obtain a high-resolution map of immune cell reconstitution after HSCT. This allows identification of biomarkers valuable for prognosis and the development of targeted therapeutic strategies to improve long-term survival in AML patients undergoing transplantation. This personalized approach may address variability in the patient response, leading to improved outcomes for high-risk AML cases. To address issues related to patient response variability, scRNA-seq aids in personalizing transplantation processes and predicting treatment outcomes based on each patient’s unique genetic profiles (Stephens et al. 2021). Recently, Obermayer et al. used scRNA-seq to study immune reconstitution dynamics up to 180 days post allo-HSCT transplantation. They found a dominance of CD8 + T-cells and identified specific molecular changes mediating allo-immune responses, such as SIRPG, LAG3, CD74, and SEMA4D. Antibody blocking experiments suggested strategies to reduce GvHD while preserving graft-versus-tumor (GvT) effects. This study contributes to understanding graft composition and informs future studies on personalized graft manipulation in allo-HSCT (Obermayer et al. 2023).

**Fig. 4:**
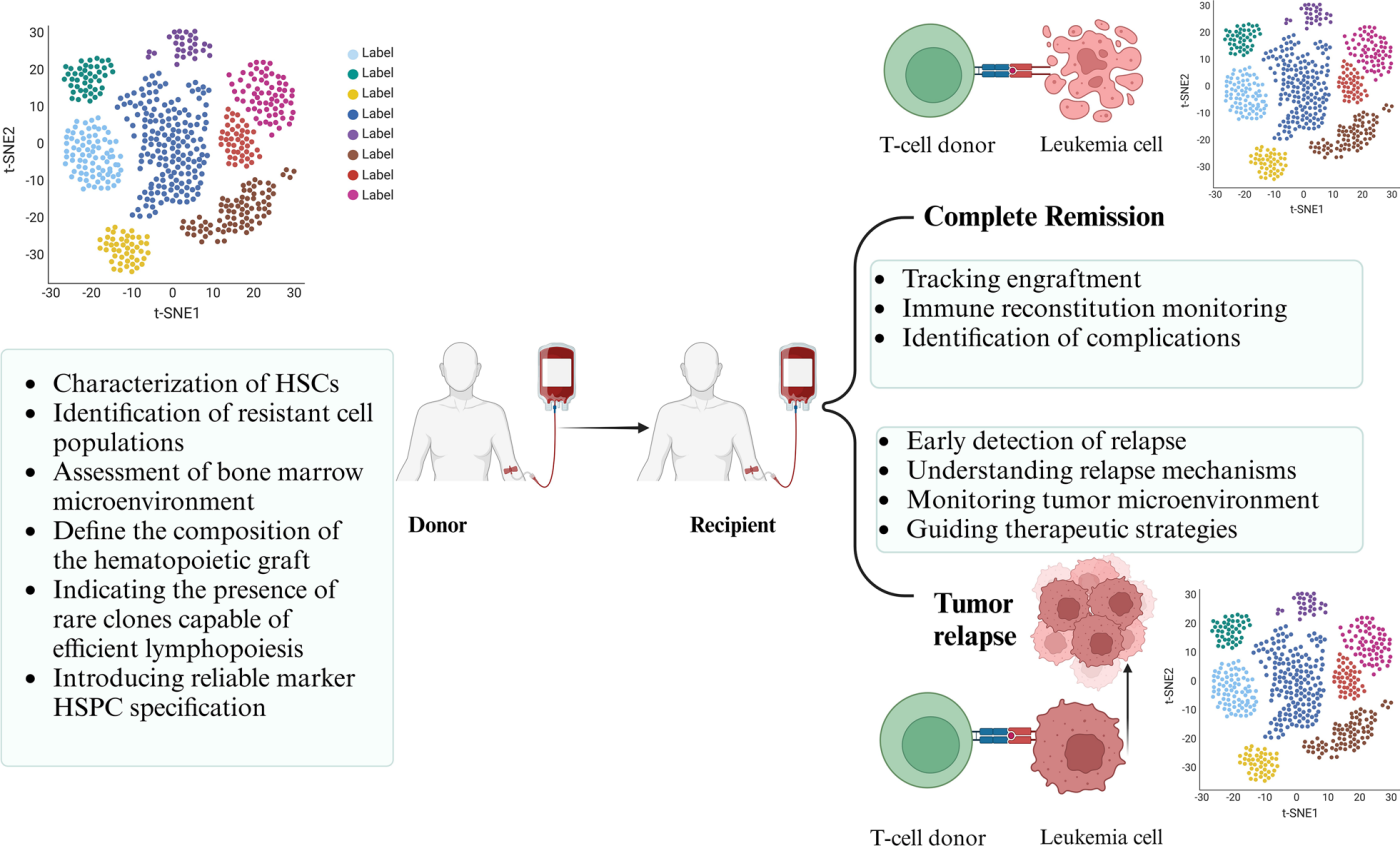
The scRNA-seq technique improves all steps of CAR-T cell therapy, from engineering to the management of adverse effects in vivo

Furthermore, scRNA-seq enables the identification and characterization of ‘off-target’ cell types that may arise during differentiation, crucial in mitigating HSCT-related complications and side effects, and addressing unexpected cellular outcomes (Ramsköld et al. 2012). By following the differentiation process through simultaneous analysis of transcripts in multiple individual cells, researchers can track transcriptional changes during the engraftment and reconstitution process following HSCT (Macosko et al. 2015). This offers single-cell insights into dynamics of the immunological system and hematopoietic recovery (Qi et al. 2019). Overall, analyzing the distinct molecular profiles of each patient’s cells based on the their unique cellular composition allows researchers to customize transplantation procedures and predict treatment outcomes (Qi et al. 2019) (Fig. 4).

### How scRNA-seq could improve the mAbs targeting AML?

ScRNA-seq plays crucial role in understanding the clonal architecture and evolutionary dynamics of AML, which is essential for developing effective treatment sterategies (Peroni et al. 2023). scRNA-seq can provide insight into the immune microenvironment linked to AML, shedding a light on the composition and function of immune cells. This knowledge deepens our understanding of the complex interactins within the immune system. Leveraging scRNA-seq data in this context not only helps optimizing the efficacy of mAbs but also enhances our ability to strategize for comprehensive AML treatment (Yeo et al. 2022). Ultimately, integrating scRNA-seq technology into AML research represents a significant advancment, offering promising developments in the design and implementaion of mAb treatments (Naldini et al. 2023).

However, significant cost and interpatient variability have limited using scRNA-seq for prospective clinical trials. To overcome these challenges, Andreadis et al. (2020) employed a genetic polymorphism-based multiplexing approach to assay peripheral blood samples from patients with relapsed or refractory AML treated in a Phase 1b study using the anti-hepatocyte growth factor mAb, Ficlatuzumab (NCT02109627). This study identified three groups of cells that expressing AML blast markers, including CD33, CD34, KIT, and HLA-DR. The hypothesis was that AML blasts inducing HGF expression might be a mechanism for resistance to anti-HGF therapy. Researchers performed unsupervised gene module analysis to identify additional biomarkers of resistance to anti-HGF therapy, and discovered 20 groups of co-regulated genes within the dataset. One module was strongly induced in non-responders compared to responders at D0, D1, and D42–44 and was highly enriched for genes related to type-1 interferon signaling and antiviral response.

These findings suggest that multiplexing using genetic polymorphisms increases the number of scRNA-seq samples that are feasible to analyze in the context of a prospective clinical trial. Gene module analysis can identify pathways as potential biomarkers of drug resistance in an unsupervised manner, assisting studies in which interpatient variability poses a challenge to the interpretation of scRNA-seq data.

### How scRNA-seq could improve CAR T-cells targeting AML?

**Fig. 5:**
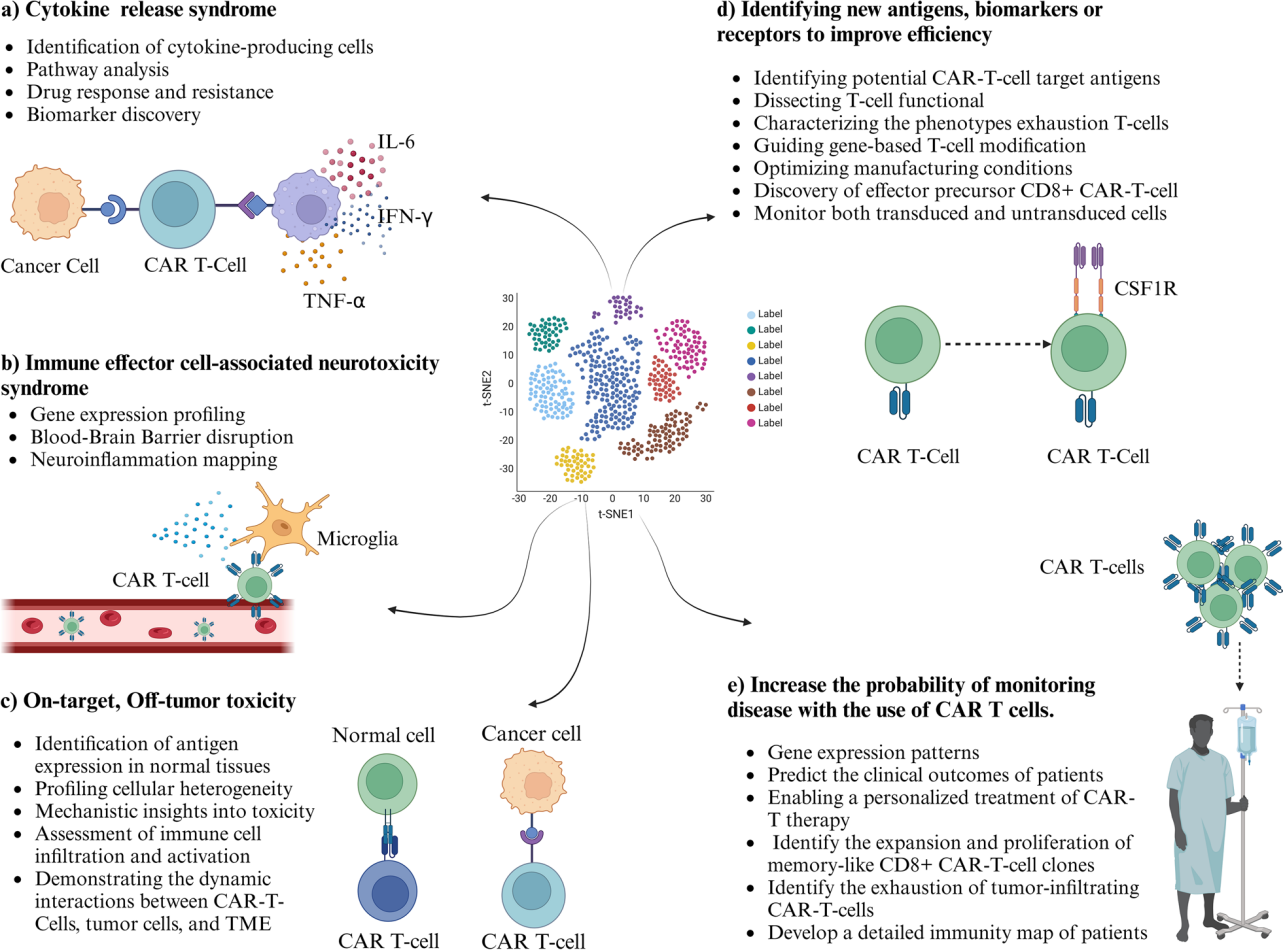
The process of developing mRNA vaccines for patients with AML and how scRNA-seq can improve their efficacy

Through scRNA-seq, a distinct peripheral CD8TEM subset was identified, showing a gene expression profile associated with NK cell functions. The study also compared findings from CAR T-cell studies, highlighting the relevance of persisting clonotypes and their gene expression patterns, suggesting the potential identification of pre-existing donor T-cell populations before transplantation (Obermayer et al. 2023). In 2023, Gottschlich et al. (Gottschlich et al. 2023) developed a scRNA-seq-based approach to identify promising antigens for CAR T-cell therapy in AML. They generated a transcriptomic atlas from publicly available datasets, which included over 28,000 healthy and malignant BM cells from 15 patients, as well as 500,000 healthy cells from nine vital human tissues. They identified two unrecognized targets for CAR T-cells in AML: colony-stimulating factor 1 receptor (CSF1R) and CD86. CAR T-cells were developed against both targets and their efficacy was tested in vitro and in vivo in patient-derived models, respectively. The results demonstrate the translational potential of an unbiased scRNA-seq-based screening approach and provide a fundation for the clinical development of CAR candidates (Gottschlich et al. 2023). Therefore, scRNA- seq can identify tumor antigen targets, such as CSF1R and CD86, as potetial targets for CAR T-cell treatment in AML (Fig. 5). These antigens showed extensive expression on AML blasts and caused minimal harm to healthy cells and organs (Atilla and Benabdellah 2023).

Nevertheless, there are challenges that remain. In a separate pilot study, CAR T-cells specifically directed against the CD123 antigen were tested in 12 people with AML that had relapsed or stopped responding to treatment. Over 90% of patients successfully underwent go through the process of making CAR T-cells, but only 25% of them had a clinical response. Additionally, 83.3% of those patients exprienced a high incidence of CRS. The study’s results suggest that therapy-induced cytokines facilitated AML blast survival through kinase signaling, leading to the development of a resistance mechanism. This mechanism includes the phenomenon known as CAR T-cell exhaustion, which is uncommon in B-cell malignancies. This shows the complexity of AML microenvironment and indiactes that using inhibitors of cytokine signaling along with CAR T-cell therapy may enhance treatment efficacy (ClinicalTrials.gov identifier: NCT03766126) (Bhagwat et al. 2024).

Briefly, ScRNA-seq is a powerful tool for development and optimization of CAR T-cell therapy. Researchers can improve the design of CAR constructs to make T cells more persistent, more effective at killing cancer cells, and better at secreting cytokines by looking at the transcriptional profiles and functional states of different types of immune cells, including T cells. This approach can also help in predicting patient responses to CAR T-cell therapy and identifying potential resistance mechanisms. Longitudinal scRNA-seq analysis allows for monitoring CAR T-cell survival, differentiation, and possible side effects over time, leting us to figure out the long term safety and efficacy of CAR T-cell therapy for AML patients (Abbas et al. 2021; Liao et al. 2024) (Fig. 5).

### How scRNA-seq could improve cancer vaccine against AML?

Samples from tumor biopsies or resections contain various cell types, which can complicate bulk RNA-seq data (Hu et al. 2018)]. Single-cell techniques can address this issue by incorporating single-cell transcriptomic profiling into neo epitope selection and vaccine manufacturing workflows. Petti et al. (Petti et al. 2019) used matched whole-genome sequencing and droplet-based scRNA-seq on samples from patients with AML, identifying abnormally differentiated tumor cells and mutation-associated transcriptional profiles (Noe et al. 2020). In a recent phase I trial, Fujii et al. used paired scRNA-seq to examine how immune systems responded to a WT1-targeted cancer vaccine in individuals who had relapsed or failed to respond to previous AML treatments. They showed that scRNA-seq could identify the specific immune cell subsets that were involved in the vaccine response, distinguishing between exhausted CD8 + T cells and those still functional post-vaccination. This in-depth study showed that the vaccine restored both innate immune cells, like invariant natural killer T (iNKT) and NK cells, and adaptive immune cells, resulting in remarkable regression of leukemia in some patients without dose-limiting side effects (Fujii et al. 2022).

Jessica Liegel et al. analyzed a DC/AML fusion vaccine in AML patients after chemotherapy-induced remission. In vaccine responders, specific gene expression signatures with upregulated immune pathways, such as IL-2, IL-7, and IL-17A, with improved T-cell diversity, along with clonal expansion, were observed. Vaccine efficacy appeared to depend on a strong immune microenvironment characterized by activated T cells and B cells that suppressed immunosuppressive elements such as TGF-β, and these signatures may serve as predictive biomarkers for achieving long-term remission in AML treatment (Liegel et al. 2022). Following this finding, Jessica Liegel et al. described a phase 1 clinical trial evaluating the safety and efficacy of a DC/AML fusion vaccine administered after allo-HSCT. Results from 19 evaluable patients showed feasibility and safety, with 14 complete remissions. Primary mechanisms included interferon signaling and T cell activation. These results suggest the potential of such vaccines to prevent relapse and maintain remission, especially under optimal conditions in the post-transplant setting (Liegel et al. 2023).

**Fig. 6:**
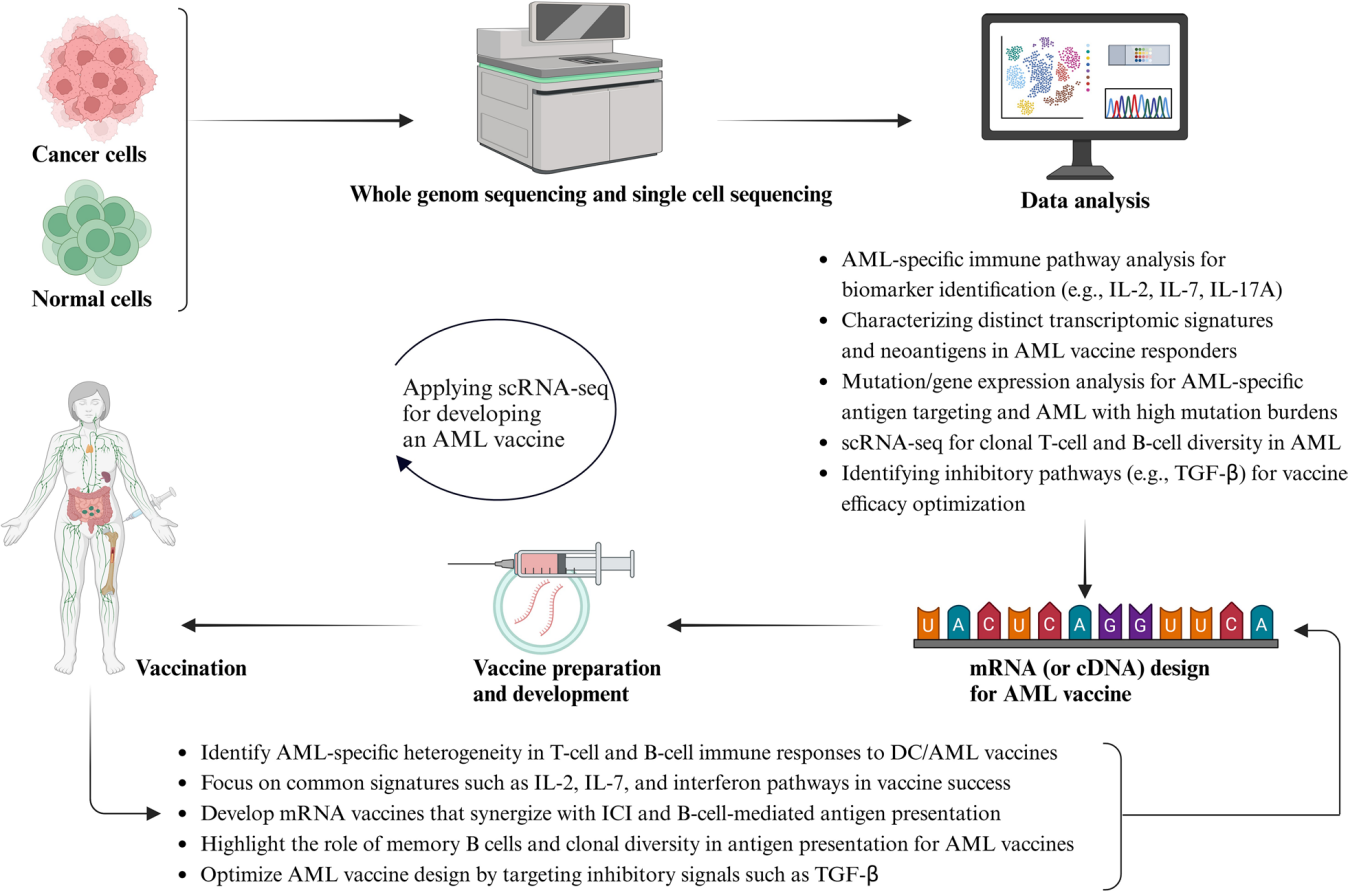
The overall mechanisms and effectors of chemotherapy resistance in AML, as well as the potential of scRNA to discover and identify these mechanisms/effectors, can improve therapeutic effects and alleviate drug resistance in vivo. For more information, refer to the text

Guo et al. investigated B-cell activation and clonal diversity within the immune microenvironment of AML using paired single-cell transcriptomics and receptor sequencing. They demonstrated a reduction in nascent B-cell populations in AML, while differentiated B-cell subsets were increased. This implies that atypical memory B cells emerged as APCs in close interaction with AML stem cells and that these interactions were associated with worse clinical outcomes. Importantly, higher B-cell clonal diversity was associated with a better response to ICB therapy. Therefore, targeting specific B-cell populations and dynamics may be a potential strategy to improve therapeutic outcomes in AML patients (Guo et al. 2024). CITE-seq combines scRNA-seq with oligonucleotide-labeled antibodies to enable simultaneous protein and transcriptome measurement at a single-cell level, enhancing neoantigen discovery pipelines. Given the variation in the therapeutic responses among pathologically identical tumors and genetically homogeneous cancer cells, scRNA-seq aids in identifying cells that respond favorably to vaccines (Lancaster et al. 2020). Therefore, by incorporating single-cell RNA sequencing into cancer research, we can gain a clearer understanding of the tumor complexities. This advanced technique allows us for the detection of specific mutations and their associated gene expressions, facilitating development of more specialized and effective personalized cancer vaccines (Fig. 6). scRNA-seq not only enhances antigen selection accuracy for effective personalized immunotherapies, but also enables monitoring of immune responses over time.

## Challenges of scRNA-seq in tumor management and possible solutions

Adopting single-cell technology not only opens up new scientific opportunities but also requires addressing specific experimental and computational/statistical challenges that are oftem similar across different single-cell applications (Lähnemann et al. 2020) (Table 1). From an experimental perspective, several standard-essential procedures are usually needed to obtain single-cell data from a biological sample (Lähnemann et al. 2020; Ziegenhain et al. 2017). These procedures include separating the cells from the target tissue, isolating and purifying the cells, creating libraries, and sequencing the data. Every stage significantly impacts the output data for subsequent analysis. For example, sample handling and preparation in scRNA-seq methods must be carefully planned to prevent unneeded stresses, which can lead to significant cellular responses and introduce artifactual transcriptional state alterations (Brink et al. 2017). The ability of multiplex various samples has been facilitated by advancemnet in microfluidics techniques for cell isolation and combinatorial indexing procedures, allowing for an increased number of cells to be sequenced in a single experiment. However, experimental steps can cause significant batch effects in subsequent analyses and contribute to technical noise. Examples of such protocols include whole genome amplification, carryover of empty droplets during library preparation, cell doublets, or dying cells (Caprioli et al. 2022).

**Table 1:** Major challenges of scRNA-seq in tumor analysis and proposed solutions

Challenges	Possible solution	Refs.
Preparation and handling of samples	Avoid unnecessary situations that cause stress	Brink et al. (2017)
Loss of histologic information	Trajectory analysis	Liu and Trapnell (2016); Jovic et al. (2022)
Loss of cellular environment	Expression map of the reference gene, in situ sequencing techniques	Liu and Trapnell (2016); Satija et al. (2015); Achim et al. (2015)
Need for separate live cells	Digestion must be carefully optimized	Ke et al. (2022)
Difficulty Identifying Gene Expression at the Lower Level	Need for low-cost, high-throughput sequencing methods	Lei et al. (2021)
scRNA-seq has only examined polyadenylated mRNAs	Random hexamer priming (SUPeR-seq), computationally "not-so-randomly" selected primers	Liu and Trapnell (2016); Chen et al. (2019c); Zhang et al. (2021); Saliba et al. (2014)
Large amount of missing data	Imputation techniques	Lähnemann et al. (2020); Das et al. (2018)
Technical noise	Noise control through reliance on inter-sample variations in the value of the ERCC readings	Brennecke et al. (2013); Ding et al. (2015)
Biological noise	Trimmed mean M values (TMM), differential expression analysis	Qi et al. (2020)
Batch effects	Include a control sample in each batch and optimize preservation techniques to allow simultaneous processing of samples	Schneider et al. 2022)
Extract meaningful data	Use a common reference or clustering method	Ginhoux et al. (2022)
Insensitivity to low abundance transcripts	?	Islam et al. (2014)
Inability to link genotype and phenotype	?	Lei et al. (2021)

While single cells can be isolated from specific areas within a cell population or tissue using micromanipulation or laser dissection techniques, these procedures are labor-intensive or require specialized equipment (Lovatt et al. 2014). Most scRNA-seq methods and all currently known high-throughput approaches involve separating tissues to produce a single-cell suspension before extracting individual cells. The loss of histological information caused by the preparation of single-cell and single-nuclei suspensions from tissues is a significant drawback of scRNA-seq, especially for solid tumors (Liu and Trapnell 2016). Trajectory analysis can aid in tracing the relationship and switching between various cell types, but other factors related to tissue enzymatic digestion, cell isolation, and preservation may alter gene expression and cell representation (Jovic et al. 2022). To overcome biases resulting from enzymatic treatments, Grindberg et al. (2013); Krishnaswami et al. (2016) developed methods for conducting RNA-seq directly on single nuclei, which can be isolated without the need for harsh protease treatments.

The original spatial context and cellular milieu of cells are lost in the majority of single-cell isolation techniques. Recently, computational techniques have been developed to determine a cell’s initial position in 3D space from its transcriptional profile, using a reference gene expression map created from available in situ data (Satija et al. 2015; Achim et al. 2015). Nature Methods has chosen spatially resolved transcriptomics technology as the Method of the Year for 2020. Understanding the composition, complexity, interactions, and roles of cells in tissues, organs, and organisms will be made possible by the emerging technologies of spatially and temporally revealing single-cell transcriptions in complex tissues and organs (Jovic et al. 2022). However, these techniques rely on the availability of spatial expression data for a panel of reference genes in the target tissue.

Alternatively, developing in situ sequencing techniques can detect and amplify RNA in the original tissue environment, although precise measurements can only be performed with a few dozen genes per cell. Using the SOLiD platform, cDNA amplicons are created, circularized, amplified by rolling circle amplification, and then sequenced by ligation in situ to sequence RNA inside unlysed cells. These in situ sequencing methods should not be confused with fluorescence in situ hybridization (FISH) techniques, which use fluorescently labeled probes to bind and identify transcripts. Although in situ sequencing techniques can determine RNA expression patterns at subcellular resolution and retain spatial information, their throughput is currently limited, and they require specialized tools that may not be widely available (Liu and Trapnell 2016).

Ultimately, the use of single-cell profiling is severely constrained by the need for separated live cells and a labor-intensive sample processing procedure. Dissociation techniques, such as hot incubation or enzymatic digestion, must be carefully optimized to produce a single-cell suspension with the minimum viability threshold indicated by the selected single-cell technology. In three crucial cases, this leads to challenges. Initially, in cases where the tissue type or sample under investigation is less viable and more sensitive, the pre-sequencing preparation method may choose a cell population that is not representative of the original sample. Second, if the study query refers to a TME topic like hypoxia, the processing technique may have hindered the transcriptome modifications brought about by the treatment situation. Thirdly, there are extra logistical hurdles because this approach requires viable single-cell suspensions. For instance, it takes a great deal of coordination and communication across several groups of individuals, from the hospital to the single-cell facility, to obtain viable cells directly from a patient following surgery (Ke et al. 2022).

Another drawback of single-cell sequencing that needs to be acknowledged is the fact that scRNA-seq implicitly suggests that not all eukaryotic cells transcribe at a consistent basal rate. Transcription occurs in pulses (Lei et al. 2021). Low-level gene expression is difficult to identify due to the technical limits of single-cell transcription level measurement; most intelligent detection methods can detect only 10–20% of genuine mRNA molecules (Islam et al. 2014; Svensson et al. 2017). Consequently, the transcriptional map cannot be fully deciphered by immediate sequencing. Furthermore, the majority of sequencing techniques are insensitive to transcripts with low abundance and are optimized for 3′ or 5′ reads (Lei et al. 2021). Since single-cell material is low, producing a larger quantity usually needs an amplification step. However, because of the amplification’s nonlinearity, which also causes bias and an unbalanced cDNA ratio in the cells, some markers cannot be amplified (Qi et al. 2020). Moreover, there is a large amount of missing data because there is considerably less sequenced material from single cells available than there is in bulk trials. Depending on the platform and depth of sequencing, missingness may be the result of technical attrition or represent an actual biological signal (e.g., changes in a gene’s expression levels). Imputation techniques that have worked better for genotype data than for transcriptome data are needed in this case (Lähnemann et al. 2020; Das et al. 2018). Conversely, a rise in the quantity of cells and attributes under investigation indicates the requirement for scalable models and techniques for data processing. The processing of high-dimensional single-cell data to make it more portable while maintaining the important biological signals of the entire dataset adds another layer of complexity (Caprioli et al. 2022).

High levels of technical noise are another effect of the small amount of input material for scRNA-seq libraries, which can obscure underlying biological variation and complicate data processing. Techniques for simulating technical differences in scRNA-seq data have been presented (Brennecke et al. 2013; Ding et al. 2015). Nonetheless, the majority of methods rely on variations in the quantity of external RNA controls consortum (ERCC) readings between samples to account for and manage technical noise in single-cell data, making them suitable only for studies that incorporate spike-in controls. Furthermore, these methods presume that during library preparation, spike-in transcripts are treated similarly to cellular RNA. Nevertheless, spiking naked RNA is not complexed with ribosomes or RNA-binding proteins, nor does it survive cell lysis. Thus, while spike-in techniques help determine the frequency and sensitivity of transcripts in an experiment, there are numerous uncontrollable reasons for variability in scRNA-seq (Liu and Trapnell 2016).

Biological noise during gene expression is major limitation in obtaining information from scRNA-seq. The high number of zero counts resulting from gene knock-out or transient expression is a significant characteristic of scRNA-seq data that could potentially mislead further analysis. The quantity of reads is correlated with a gene expression level, but assessing confounding variables is challenging. Trimmed mean M values (TMM) and differential expression analysis for sequence count data (DESeq) are commonly used methods for between-sample normalization. Both approaches, based on the weighted mean or median of the samples, exclude certain genes, but struggle when a high number of zero counts are recorded. The number of reported readings is also impacted by confounding variables like biological factors and technological noise, in addition to normalization (Qi et al. 2020).

Another point to keep in mind is that single-cell experiments introduce more noise than bulk sample sequencing because each individual cell can be thought of as its own biological duplicate. Technical artifacts resulting from processing data at different times or by different people intensify the experimental batch effects due to the technology’s great sensitivity and requirement for rapid profiling. Two ways to combat these batch effects are to include a control sample in each batch that can be used to computationally compensate for technical artifacts, or to optimize preservation techniques to allow samples to be processed simultaneously. New advancements in machine-free single-cell profiling through kits could make this method more widely available and decrease some of the associated technical and logistical difficulties (Schneider et al. 2022). Some of these limitations can be addressed by using alternate techniques, such as single-nucleus RNA sequencing and flash-freezing for nucleus isolation, which have been demonstrated to be comparable to scRNA-seq in single-cell analysis of cryopreserved samples. However, such techniques may still not be appropriate for the investigation of more susceptible cell types with lower viability or a specific TME component. As a result, every stage of the pre-sequencing protocol needs to be optimized to ensure that the sample can still shed light on the current research subject (Slyper et al. 2020; Denisenko et al. 2020).

As with many other large datasets, one potential pitfall with scRNA-seq is trying to extract meaningful and interesting data. Since scRNA-seq mostly depends on cell subset annotation, it is difficult to recreate and generate biological validation in the absence of a common reference or clustering method. For example, different definitions of cell states and subsets in different studies lead to significant misunderstandings in the interpretation of results. This problem has recently been highlighted in a dendritic cell study example (Ginhoux et al. 2022). Furthermore, scRNA-seq by itself is unable to link genotype and phenotype, highlighting the necessity for low-cost, high-throughput sequencing methods in order to map the entire tumor tissue landscape (Lei et al. 2021).

The majority of research on scRNA-seq has only examined polyadenylated mRNAs. Poly-T priming, which captures only polyadenylated transcripts, is used in almost all reported scRNA-seq procedures to isolate cellular RNA. Therefore, non-polyadenylated transcript types, such as bacterial RNA and regulatory non-coding RNAs (like microRNAs, lncRNAs, or circular RNAs), cannot be studied using current methodologies. Some approaches are currently being developed to capture poly(A)- and poly(A) + transcripts stimultaneously (Liu and Trapnell 2016; Chen et al. 2019c). Random hexamer priming is example of this approach that act based on random primers for single-cell poly(A)-independent RNA sequencing (SUPeR-seq), allows for the detection of poly(A) + and poly(A)-RNA in single cells. However, due to its rarity and limited application in tumor research, further developments in this field are still needed (Zhang et al. 2021). Additional approaches involve the use of computationally "not-so-randomly" selected primers, which can be employed to detect poly(A) + and poly(A)-species, whereas for ribosomal RNA51 depletion, by integrating these alternate priming methods into current scRNA-seq technologies, the scale and utility of scRNA-seq can be increased, allowing for the exploration of greater variety of transcript types (Saliba et al. 2014).

In the field of AML research, scRNAseq encounters several challenges. Robust analytical performance is essential for real-time clinical inquiries (e.g., critical care alerts) and supporting research computational requirements (e.g., omics data analysis). To achieve accuracy, one must address the predicted heterogeneity and poor quality of data while also adopting a paradigm shift towards data-driven analytics (Soudris et al. 2015). From a statistical perspective, a significant issue is that the sample size is sometimes considerably smaller than the number of variables in certain genomic datasets. Working with omics data presents significant challenges in sequence alignment, which can lead to the spread of additional noise as more implications emerge from the data. Furthermore, AML research presents unique challenges. Registry-based data about diagnosis, treatment administered, and clinical outcomes exhibit significant shortcomings, as evidenced by the Surveillance Epidemiology and End Results (SEER)—Medicare database, which indicates that up to 50% of AML cases were underreported (Craig et al. 2012). Practical information regarding the clinical outcomes of AML patients is insufficient. When such data are accessible, the observations and conclusions generated from them may seem incompatible with those obtained from published clinical trial data (Juliusson et al. 2012). This situation requires modification to mitigate the effects on some AML investigations utilizing this data.

On the other hand, challenges persist to a certain extent due to inadequate coverage, limited access to complete RNA sequences, RNA modifications, and inappropriate algorithms for mutation detection, data normalization, differential gene expression analysis, dimensionality reduction, and mutational heterogeneity of blast cells (Hwang et al. 2018). Although there is scope for advancement, sc-seq has established its particularity and significance in numerous investigations, indicating high future potential and requiring additional optimization. Significant discoveries and explorations are ongoing, and continued utilization and development of new technologies will advance our goal of optimal solutions for AML.

## Artificial intelligence for supporting scRNA-seq

Artificial intelligence (AI) is the term used to describe computer systems that mimic human intellect, including learning and problem-solving abilities, and have access to large amounts of data (Shouval et al. 2021). While AI technologies are increasingly integrating into everyday life, medicine has taken a leading role in storing, retrieving, and discovering patient data. These technologies have become essential in recent times because of an increase in chronic diseases, massive amounts of data, and pandemics spreading throughout the world. Various applications of AI for healthcare-based areas include analysis of patient behavior, clinical decision support, and medical image analysis. One of the most exciting areas considered is diagnostic hematology (Briganti and Moine 2020; Gedefaw et al. 2023). AI has been applied to hematology since 1994, when three knowledge-based systems were implemented in Europe to evaluate immunophenotyping, BM reports, and complete blood counts (CBC) in patients with leukemia (Zini 2005). Machine learning (ML) is a subtype of AI that allows computers to learn without following predetermined guidelines (Wong et al. 2022). ML has significant subtypes, namely: reinforcement, unsupervised learning, and supervised learning. Unsupervised ML finds patterns or groups in the data, while supervised ML uses a labeled training set to predict outcomes. By combining supervised and unsupervised learning, reinforcement machine learning maximizes accuracy through trial and error. However, a large amount of data is required for ML systems to learn efficiently (Shouval et al. 2021). AI has been cast into an important role in diagnosis, particularly in the analysis of flow cytometry data, helping to increase sensitivity and precision in identifying leukemic cells. AI-driven methods have indeed shown excellent performances for leukemia subtyping. Models like ResNet-50 achieve 94.6% sensitivity for AML and 98.2% for B-ALL. AI could accelerate diagnosis in AML without forfeiting compliance with frameworks such as the FATF principles or the EU AI Act. These frameworks aim to ensure transparency and fairness of AI systems, despite challenges related to biases, ethical concerns, and interpretability. AI considerably reduces the time needed for cells classification of compared to manual methods, although limitations persist, such as small datasets and difficulty in identifying rare cells. These advances underscore the need for ethical deployment and strong regulatory oversight if such technologies are to be sustainably integrated into clinical workflows (Cheng et al. 2024). AI’s application extends beyond diagnosis to prognosis, where it supports personalized medicine by predicting outcomes based on individual clinical and genetic data. ML models can provide personalized survival predictions, empowering clinicians to make well-informed treatment decisions for AML patients (Awada et al. 2022). AI also enable the identification of therapeutic targets, integrating genomic data with drug screening results to develop personalized treatment options, (Gimeno et al. 2022). However, biases still exist, especially within data sets, which may affect individual outcomes based on age, sex, or other characteristics, and thus diverse, representative training datasets are imperative (Sadafi et al. 2023).

Several AI and ML techniques can be used to solve these problems in high-dimensional scRNA-seq data analysis. These include getting rid of batch effects, filtering out noise, and, most importantly, finding small biological patterns. For instance, unlike traditional methods like principal component analysis (PCA), which reduce dimensionality by focusing on principal components, advanced AI models such as iMAP and BERMUDA correct batch effects to classify distinct cellular subtypes with higher resolution. This will be crucial in AML, as the differentiation between malignant and non-malignant cells is essential for optimizing therapy (Wang et al. 2021). Massive datasets and rapid advances in computation and ML have spurred the development of new machine intelligence systems for cell fate analysis and prediction in scRNA-seq data. These methods can be used to investigate generative adversarial network methods for estimating high-dimensional data distributions in the single-cell gene expression space, and to identify suitable supervised deep learning architectures and training algorithms for scRNA-seq data (Marouf et al. 2020). scRNA-seq technique generates enormous amounts of data, and instead of revealing the right answer, conjecture may result from biased analysis, technical noise, and stochastic behavior of the data. The most promising approach to solving these issues may lie in AI and ML (Goldsmith et al. 2022). As an example, AML researchers have used these RF classifiers to identify different cellular hierarchies, such as HSC-like and GMP-like AML cells associated with prognosis and drug resistance. The reason could be that AI is capable of processing complex scRNA-seq data sets, an area where other traditional methods often fall short (Galen et al. 2019). Recently, a number of studies have been conducted using AI-based single-cell sequencing analysis for hematological diseases (Lei et al. 2021). Artificial neural network (ANN) models performed best (accuracy: 82.8%) in screening for hematologic malignancies compared to other ML models out of more than eight AI-based ML models evaluated (Syed-Abdul et al. 2020).

Based on gene expression profiles, the researchers created a deep learning (DL) model called Deep Myelodysplastic Syndromes (DeepMDS) that was able to predict patient outcomes with high accuracy. They found that DeepMDS was more accurate than previous prediction models and discovered a number of unique biomarkers associated with disease progression (Awada et al. 2022). Nicora et al. (2020) developed a method for improving the reliability of ML predictions within the context of scRNA-seq data. The authors furthered classification capability in tumor and normal cells of AML patients by proposing a measure of reliability that assesses the extent to which new samples match the distribution of the training data. This approach has become extremely useful in monitoring disease progress and treatment response by quantifying tumor cells over time. Nonetheless, the study highlighted challenges such as dataset-specific biases and the computational expense of attribute-by-attribute assessments, which could hinder scalability and adaptability to diverse patient populations. Building on this, Asimomitis et al. employed DL techniques to predict both phenotypic and genotypic profiles in AML using scRNA-seq data. Their binary classification model achieved an impressive accuracy of 98% in distinguishing malignant cells from wild-type cells. They further extended this work to a multi-class, multi-label framework that allowed the prediction of specific mutations and chromosomal abnormalities. They also identified critical genes in AML-related pathways like IL-2/STAT5 and NF-kB signaling. However, the approach relies very strongly on high-quality, curated datasets and requires significant computational resources, which may limit its use in resource-poor environments (Asimomitis et al. 2023). The integration of scRNA-seq with molecular genetics and flow cytometry, could further increase the precision of classification and hence AML patient-specific treatment strategies. This study also discussed the value of ML in driving MRD evaluation forward by improving risk stratification. However, several barriers are existed to clinical implementation: most of the studies were retrospective; extensive datasets are required; and there is a lack of regulatory frameworks that would ensure the safety of application. The authors underlined that prospective validation and collaboration are urgently needed in order to standardize machine learning applications across diverse clinical settings (Eckardt et al. 2020).

The AI also enhances in the identification of rare cell populations through scRNA-seq. Variational autoencoders, like those used in tools such as ScGen, model perturbation responses to help understand how AML cells react to therapeutic stress. These models are particularly useful in identifying subpopulations with resistance mechanisms (Gui et al. 2023). AI has the potential to assist patients with AML in selecting the best treatment options. Target analysis has identified potential therapeutic protein targets, and various AI algorithms have been used to eliminate treatments and identify viable therapeutic options (Gedefaw et al. 2023). For instance, predictive models utilizing both bulk RNA-seq and scRNA-seq data have been developed to predict treatment outcomes more accurately than pervious methods. AI models like SAVER-X and scVI serve as robust platforms, integrating data from multiple platforms with a minimal technical noise, overcoming critical limitations in AML research. In addition, by providing insights into the molecular mechanisms underlying disease progression, AI-based single-cell sequencing analysis has the potential to improve the diagnosis and treatment of AML (Moran-Sanchez et al. 2021; Li et al. 2020). Jiao et al. used a multi-level convolutional neural network (MulCNN) to extract important features through multi-scale convolution while filtering noise, creating a unique single-cell gene expression profile (Jiao et al. 2023). Addressing the challenge of batch effects, another innovative DL-based technique called BERMUDA (batch effect removal using deep autoencoders) provided higher resolution of cellular subtypes. By combining various cell compositions with multiple batches of scRNA-seq data. Wang et al. found that this new model outperformed previous techniques for batch effect removal and cell type differentiation between in numerous datasets, including actual scRNA-seq datasets, (Wang et al. 2019). A recent advancement in this area is single-cell variational inference (scVI), a scalable system for scRNA-seq data analysis (Lopez et al. 2018).

Another ML-based model, known as scGen, combines variational autoencoders with latent space vector arithmetic to accurately model infection and perturbation responses in cells across studies, species, and cell types. This will improve our knowledge of how to screen for perturbation responses in the context of disease and drug treatment (Mohammad Lotfollahi FAWaFJT 2019). The more robust SAVER-X framework, which trains a deep neural network across multiple research designs and applies this model to new data to capture common biological information across diverse datasets, was recently developed by merging deep autoencoders with a Bayesian model (Wang et al. 2019). Together, these improvements show how AI can expand scRNA-seq analysis, providing new information about TME, heterogeneity, and resistance mechanisms. Such integration is particularly essential in AML, as the accurate data interpretation directly impacts treatment outcomes (Gui et al. 2023). Researchers requremore effective methods to manage high-dimensional transcriptome data. Fortunately, AI can adequately address the growing need for transcriptome analysis. AI techniques can enhance decision making and discovery with minimal human assistance. By implementing AI-based frameworks, we can also uncover hidden information and obtain more valuable results. AI can facilitate cross-platform data integration and batch effect elimination (Liu et al. 2021). In fact, AI-assisted transcriptome analysis accelerates the process of uncovering the microenvironment and tumor heterogeneity while improving the prediction of immunotherapy response. Therefore, future AI/ML-based systems must tackle current challenges and provide more accurate, copmerhensive, and reliable diagnostic and predictive algorithms using typical large scRNA-seq datasets (Gedefaw et al. 2023). Overall, as the cornerstone of analyzing high-resolution RNA sequencing data, cell annotation faces challenges such as high sparsity and noise in data sets, as well as batch effects. AI technologies have entered the field of developing cell annotation algorithms, showing excellent performance. While there have been several algorithm reviews in this area, they have primarily focused on evaluating algorithmic performance rather than presenting of computational solutions. In light of these facts, AI is changing the management of AML in diagnosis, prognosis, and personalization of treatments. However, biases in data, interpretability of models, and lack of strong regulatory mechanisms are major concerns that need urgent attention. The integration of AI into clinical oncology holds immense promise, but its full potential will only be realized with ethical deployment, rigorous validation, and commitment to equitable patient care.

## Conclusion

This review discusses the intricate relationship between therapeutic resistance mechanisms, allo-HSCT-asscciated issues, and treatment outcomes in AML, emphasizing discoveries derived from scRNA-seq. Despite advancements in standard therapy regimens and targeted therapies, drug resistance and relapse remain significant barriers to improving AML prognosis. The integration of scRNA-seq into AML research has provided insights to the mechanisms causing therapeutic failure, revealing prospective therapeutic targets, as well as presenting new pathways to overcome resistance. Furthermore, differentiation therapies and epigenetic modulators exhibit potential as alternate approaches to overcome resistance and improve therapeutic efficacy. By revealing the molecular and cellular heterogeneity of AML, scRNA-seq provides a revolutionary methodology for the development of more precise and efficacious therapy strategies, ultimately aiming to diminish recurrence rates and enhance long-term survival for AML patients. In conclusion, scRNA-seq reveals common transcriptome/pathways present in different AML genetic subtypes enabling the development of a profile for prognosis and treatment of AML. At the same time, scRNA-seq indicate the distinct differences were identified in AML-associated sub-clone as well as make-up in the BM microenvironment that distinguished samples with relapse from samples with complete remission. scRNA-seq together with associated computational tools and AI uncovered the presence of patient-specific signature, which is important for diagnostic and monitoring purposes that may improve therapeutic potential of existing therapies against AML, while identifying AML-blast specific gene signatures direct development of cancer vaccine or engineered CAR T-cells with satisfactory efficacy in the clinic in the future.

## Supplementary Information

Additional file 1.

## Data Availability

No datasets were generated or analysed during the current study.
