# Supplementary material for: Single cell RNA sequencing improves the next generation of approaches to AML treatment: challenges and perspectives
Source: Mol Med. 2025 Jan 30;31:33. doi: 10.1186/s10020-025-01085-w (PMC11783831; doi:10.1186/s10020-025-01085-w)
Supplement: Supplementary file 1 — Additional file 1. [file 10020_2025_1085_MOESM1_ESM.docx]

**Table**. Clinical trials with antibodies in AML treatment

| **Target** | **Type** | **Status** | **Efficacy** | **NCT** |
| --- | --- | --- | --- | --- |
| CD33xCD3 | Bi-mAb (emerfetamab) | Phase 1 | - 44% with blast reduction - 22% with >50% blast reduction | NCT03224819 |
| CD47 | mAb (magrolimab) | Ongoing 1b | ORR 69%: 50% CR or CRi, 13% PR and 31% SD | NCT03248479 |
| CTLA-4 | mAb (nivolumab) | Phase 1 | Durable response(>1year): 4/22 | NCT01822509 |
| PD-1 | mAb | Phase 2 | ORR: 33%  mOS: 10.6 months | NCT02397720 |
| CD33 | mAb | Phase 2 | 69% remission | NCT02575963 |
| CD33 | mAb | Phase 1 | CR/CRi 11.4% | NCT02520427 |
| CD123 | mAb | Phase 1 | N/A | NCT03647800 |
| CD123 | mAb | Phase 1 | CR/CRi: 23% | NCT02730312 |
| CD123 | mAb | Phase 1/2 | CR: 1/12, CRi: 3/12 | NCT03386513 |
| CD123 | mAb | Phase 1/2 | ORR 13.6%, CR 11.7% | NCT02152956 |
| CD45 | mAb | Phase 1 | OS at 1.8 years: 53% | NCT01300572 |
| CD70 | mAb | Phase 1//2 | CR/CRi: 83% | NCT03030612 |
| CD47 | mAb | Phase 1 | NA | [NCT02641002](https://clinicaltrials.gov/ct2/show/NCT02641002) |
| CD33 | mAb | Phase 3 | CR: 47%case-58%control  OS:73%case-69%control | NCT00893399 |
